# Genetic Testing for Lynch Syndrome in Individuals Newly Diagnosed with Colorectal Cancer to Reduce Morbidity and Mortality from Colorectal Cancer in Their Relatives

**DOI:** 10.1371/currents.RRN1246

**Published:** 2011-07-06

**Authors:** Ralph Coates, Marc Williams, Stephanie Melillo, Jim Gudgeon

**Affiliations:** ^*^Associate Director for Science, Public Health Surveillance Program Office, Centers for Disease Control and Prevention; ^†^Director, Intermountain Healthcare Clinical Genetics Institute; ^‡^Office of Public Health Genomics, Centers for Disease Control and Prevention and ^§^Health services research, focus in clinical genetics

## Abstract

Individuals with Lynch syndrome, sometimes referred to as hereditary non-polyposis colorectal cancer (HNPCC), have an increased risk of developing colorectal cancer (CRC) as well as other cancers. The increased risk is due to inherited mutations in mismatch repair (MMR) genes, which reduce the ability of cells to repair DNA damage. Screening for Lynch syndrome in individuals newly diagnosed with colorectal cancer has been proposed as part of a strategy that combines tests and interventions to reduce the risk of colorectal cancer in the relatives of the colorectal cancer patients with Lynch Syndrome.

## **Clinical Scenario**

Screening tests on tumor specimens and genetic testing for germ line mutations in mismatch repair (MMR) genes of individuals newly diagnosed with colorectal cancer to identify patients with Lynch syndrome to reduce morbidity and mortality from Lynch syndrome in their relatives [1] [2] [3].  

## **Test Description**

**Screening tests: **Before genetic testing for MMR mutations, preliminary screening tests on tumor tissue may be considered in patients with colorectal cancer. This screening improves efficiency and reduces costs by identifying patients who would most likely have MMR mutations [1] [2] [3]. One or several of the following tests may be offered:  

- **Microsatellite instability (MSI) testing** assesses the stability of small DNA sequences in tumors, a measure of the integrity of DNA repair mechanisms. Patients with a high instability score from MSI testing can then be offered DNA sequencing for the four most common MMR genes associated with Lynch syndrome (*MLH1, MSH2, MSH6, *and *PMS2*) [1] [2] [3].
- **Immunohistochemical (IHC) staining** tests tumor tissue for the presence of the proteins produced by four MMR genes. The absence of staining for one or more of the proteins indicates the high likelihood of the presence of a mutation in one of the MMR genes which can be due either to a germ line mutation or a somatic abnormality limited to the tumor. The absence of staining among the four specific proteins is used to identify specific genes for sequencing [1] [2] [3].

      ***BRAF (V600E) *and/or *MLH1 *promoter hypermethylation testing** may be performed on cases that have no IHC staining for MLH1, as a substantial portion of tumors that lack staining for the MLH1 protein have a somatic mutation in *BRAF* (V600E)or *MLH1 *promoter hypermethylation, neither of which is commonly associated with Lynch syndrome.  Patients who do not have the *BRAF* mutation or *MLH1 *promoter hypermethylation, or have neither, are then offered DNA analysis of the *MLH1* gene [1] [2] [3].

**Diagnostic test: ** 

DNA sequencing, and possibly analysis for copy number variants, of the 4 major MMR genes (*MLH1, MSH2, MSH6, *and *PMS2*) associated with Lynch syndrome, is the practice standard test for diagnosing the syndrome after a positive screening test [1] [2] [3].   

## **Public Health Importance**

Of the approximately 143,000 individuals diagnosed with colorectal cancer each year, about 3% (4,250) have Lynch syndrome. Approximately 20 to 65% of individuals with Lynch syndrome develop colorectal cancer during their lifetimes, whereas lifetime risk in the general population is approximately 5%. In addition, about half of the first-degree biological relatives of those colorectal cancer patients with Lynch syndrome, about 8,000 individuals, also have Lynch syndrome mutation and are at increased risk of developing a Lynch syndrome-associated cancer. Screening and treatment for colorectal cancer has been found to substantially reduce the risk of developing colorectal cancer in individuals with Lynch syndrome as well as in the general population. Identifying Lynch syndrome in newly diagnosed colorectal patients, offering testing to relatives of patients with Lynch, finding those relatives who have Lynch syndrome before they develop cancer, and offering screening to those relatives could reduce their risk of colorectal cancer.  Potentially more than a thousand cases of colorectal cancer could be prevented each year if all individuals with Lynch were identified, screened, and treated appropriately [1] [2] [3] [4] [5] [6] [7] [8].  

**Systematic Evidence Reviews**

Agency for Healthcare Research and Quality, Evidence Report/Technology Assessment (Bonis) http://www.ahrq.gov/clinic/tp/hnpcctp.htm

Evaluation of Genomic Applications in Practice and Prevention Supplemental Evidence Review (Palomaki) http://www.egappreviews.org/docs/EGAPPWG-LynchRev.pdf  

Recommendations by an Independent Group*

The Evaluation of Genomic Applications in Practice and Prevention Working Group recommended offering genetic testing for Lynch syndrome in individuals newly diagnosed with colorectal cancer to reduce morbidity and mortality in relatives. (EGAPP) http://www.egappreviews.org/docs/EGAPPWG-LynchRec.pdf   

**Guidelines by Professional Groups**

The American College of Gastroenterology recommends that colorectal cancer patients who have a family history of colorectal cancer that indicates increased risk of HNPCC according to either Bethesda or Amsterdam family history criteria have tumors screened by MSI or IHC [9]. 

The National Comprehensive Cancer Network recommends tumor screening and/or genetic testing for individuals with family histories meeting the Bethesda or Amsterdam criteria, for individuals diagnosed with endometrial cancer before age 50, and individuals with a family member with Lynch syndrome [10]. 

A task force with representatives from several medical professions and the American Cancer Society recommends colonoscopy every 1 to 2 years beginning at age 20 to 25 for individuals who have a clinical or genetic diagnosis of HNPCC or are at increased risk of HNPCC based on the modified Bethesda criteria [11].     

## **Evidence Overview**

***Analytic validity***

: The accuracy and reliability of the tests in detecting the genetic changes of interest. 

Based on evidence reviews, the EGAPP Working Group reported that overall the analytic validity of the tests was high although there were gaps in research on analytic validity and proficiency testing, as described below [1] [2] [3]: 

- **MMR**: DNA sequencing of 4 MMR genes (*MLH1, MSH2, MSH6, *and *PMS2*) is the practice standard, but actual performance is difficult to estimate, and it is not known if laboratory proficiency testing will be an adequate validity measure. [1] [2] [3].
- **MSI**: Testing is offered by many laboratories that participate in proficiency testing programs, and performance in such testing programs is high so adherence to best practices may provide valid testing [1] [2] [3].
- **IHC**: IHC proficiency testing is offered for other proteins, but not specifically for MMR gene proteins (1-3, 19)[1] [2] [3] [12].
- ***BRAF***: Given that this test is to identify a single mutation and proficiency testing for some other single mutations has been high, analytic validity is likely to be high [1] [2] [3].

***Clinical validity***: The accuracy and reliability of the test in identifying patients with the disorder. 

Based on the evidence reviews, the EGAPP working group reported that there was adequate evidence of clinical validity for the preliminary tests, although evidence varied and research gaps were identified on which tests and which combinations perform best and on the use of family history with tests, as described below [1] [2] [3]. 

- **MMR**: DNA sequencing of 4 MMR genes (*MLH1, MSH2, MSH6, *and *PMS2*) is the current standard for diagnosing patients with Lynch syndrome, although research may identify additional MMR genes. Lifetime risk of colorectal cancer among individuals with Lynch syndrome is approximately 20 to 65% [1] [2] [3] [7] [8].
- **MSI**:  Studies enrolling a total of 150 patients with Lynch syndrome and using a variety of MSI methods found that high MSI score test results were adequately sensitive and specific in identifying individuals who had tested positive for some MMR genes [1] [2] [3].
- **IHC**: Studies with a total of 149 patients found that IHC testing was adequately sensitive and specific in identifying individuals who had tested positive for some MMR genes [1] [2] [3].
- ***BRAF***: Multiple studies have found *BRAF* mutation testing to be highly sensitive in identifying CRC tumor tissue abnormal for the MLH1 protein due to somatic changes. Thus, it is a highly effective test for ruling out the presence of Lynch Syndrome [1] [2] [3].

Research has shown that concordance between IHC and MSI test results varies [12] [13]. 

The EGAPP Working Group found insufficient evidence to recommend any one of the screening-diagnostic testing combinations or sequences over the others [1] [2] [3], and there appears to be no consensus among practitioners on a best practice.  

***Clinical utility***:The possibility that implementing a screening/testing and intervention program for Lynch Syndrome will lead to improved health. 

Based on the evidence reviews, the EGAPP working group reported that there was adequate evidence from research that more than about 90% of relatives of patients with Lynch would consent to genetic testing and that more than half of those who were identified as having Lynch syndrome began screening with colonoscopy, beginning at age 20-25. A single study of relatives at high risk provides evidence that screening colonoscopy results in an approximately 60% reduction in the incidence of colorectal cancer. Harms appeared to be minimal in comparison with benefits. However, additional research was needed on the overall strategy and on each step from offering genetic testing to patients through studying the long term health benefits to relatives. Additional cost-benefit analyses are also needed. Screening or prophylactic surgery for prevention of other Lynch syndrome associated cancers (particularly endometrial) has not been fully assessed for utility [1] [2] [3].  

The EGAPP Working Group found insufficient evidence of benefit to CRC patient from modification of clinical management options based on testing for Lynch [1] [2] [3]. The American College of Gastroenterology found moderate quality evidence for their recommendation that colorectal cancer patients with Lynch syndrome undergo colonoscopy every 2 years beginning at age 20-25 and then annually after age 40 [9]. 

A cost-effectiveness analysis has reported that a testing strategy using IHC as the preliminary test for individuals newly diagnosed with colorectal cancer has an incremental cost-effectiveness ratio of < $25,000 per life-year saved compared to no testing for a Lynch syndrome [14].  

Some researchers, practitioners, and members of the public have raised questions about how best to implement testing beyond research settings, how feasible it is, and how potential harms from implementation might be addressed [15] [16] [17].  These questions raise issues about the clinical utility and cost effectiveness of testing in common practice. 

Research is continuing on the potential utility of screening CRC patients for Lynch syndrome to assist in treatment decisions [18] [19].    

**Contextual issues:** Including clinical alternatives to genetic testing, practice, ethical, legal, social issues. 

The EGAPP Working Group, based on the evidence reviews, found that methods using family history, either the Amsterdam or Bethesda criteria, to identify patients with Lynch syndrome produced inconsistent results and identified a lower percentage of patients with Lynch syndrome than did tumor-based screening protocols.  They also recommended informed consent for preliminary testing of patients, noted that studies suggest adverse psychosocial outcomes should be minimal, and stated that resource requirements appeared to be justified by willingness of relatives to participate and health benefits for relatives [1] [2] [3]. 

A report suggests that more research is needed on psychosocial issues because of evidence that some subgroups are more vulnerable to testing-related stress [20].

Lynch syndrome is associated with increased risk of a number of other cancers, including cancers of the endometrium, stomach, small intestine, bladder, brain, kidney, and biliary tract, and individuals diagnosed with Lynch syndrome may be monitored by their clinicians for those cancers as well (2-3).

The Amsterdam criteria may be used to screen individuals for presnce of Lynch syndrome based on their family history of Lynch syndrome-asociated cancers (3).  The revised Amsterdam criteria include the following: at least 3 relatives with Lynch-associated cancer, one should be a 1st degree relative of the other two, at least 2 generations with Lynch-associated cancers, at least 1 diagnosed before age 50, excluding familial adenomatous polyposis, and with tumors verified by pathologic examination (3). The Bethesda criteria may be used to identify patients with colorectal cancer who could benefit from genetic testing for MMR gene mutations (3). The revised Bethesda criteria include the following: colorectal cancer diagnosed in a patient younger than age 50, presence of synchronous, metachronous, colorectal or other Lynch-associated cancers regardless of age, colorectal cancer with the MSI-H-like histology diagnosed in a patient age less than 60, colorectal cancer diagnosed in a pateint with one or more 1st degree relatives with Lynch syndrome-related tumor -- with one of the cancers being diagnosed under age 50, or colorectal cancer in a patient with two or more 1st or 2nd degree relatiaves with Lynch syndrome-associated cancers regardless of age.

Given the variety of screening approaches, including scoring of histopathological characteristics, family history criteria, and inconsistencies in the recommendations from various groups, practicing clinicians face multiple options for Lynch syndrome screening. At present, there is limited published research on how to effectively implement testing in routine clinical practice and on the benefits and costs of implementation [15] [16] [17] [21].  

## **Additional Links**

Epidemiologic research on genes associated with Lynch, HNPCC:    http://hugenavigator.net/HuGENavigator/phenoPedia.do?firstQuery=Colorectal Neoplasms, Hereditary Nonpolyposis&cuiID=C0009405  &typeSubmit=GO&check=y&which=2&pubOrderType=pubD/

http://hugenavigator.net/HuGENavigator/phenoPedia.do?firstQuery=Lynch Syndrome II&cuiID=C1333991  &typeSubmit=GO&check=y&which=2&pubOrderType=pubD/  

**Acknowledgements:** 

We would like to thank Katherine Kolor, PhD, MS, Office of Public Health Genomics, Centers for Disease Control and Prevention, Atlanta, GA and Cecelia A. Bellcross, Ph.D. MS. Emory University, Atlanta, GA for reviewing and commenting on this brief review prior to submission for publication...  

**Funding Information:** 

Ralph Coates and Stephanie Melillo were supported by their employer, the Centers for Diseae Control and Prevention, Atlnata, GA, USA. Marc Williams and Jim Gudgeon were supported by their employer, Intermountain Healthcare, Salt Lake City, UT, USA.  

**Competing Interests:** 

The authors have declared that no competing interests exist. 

*Independent groups include the Advisory Committee on Heritable Disorders in Newborns and Children, the Evaluation of Genomic Applications in Practice and Prevention (EGAPP) Working Group, the United Kingdom's National Institute for Health and Clinical Excellence, and the US Preventive Services Task Force (USPSTF).
